# Phenomenological Skepticism Reconsidered: A Husserlian Answer to Dennett’s Challenge

**DOI:** 10.3389/fpsyg.2020.02058

**Published:** 2020-09-04

**Authors:** Jaakko Belt

**Affiliations:** Unit of History, Philosophy and Literature, Faculty of Social Sciences (SOC), Tampere University, Tampere, Finland

**Keywords:** Husserl, Dennett, phenomenology, methodology, reflection, introspection, skepticism, first-person methods

## Abstract

There is a long-standing tradition of questioning the viability and scientificity of first-person methods. Husserlian reflective methodology, in particular, has been challenged on the basis of its perceived inability to meet the standards of objectivity and reliability, leading to what has been called “phenomenological skepticism” (Roy, 2007). In this article, I reassess this line of objection by outlining Daniel C. Dennett’s empirically driven skepticism and reconstructing his methodological arguments against Husserlian phenomenology. His ensuing phenomenological skepticism is divided into strong skepticism and categorical and gradual versions of weak skepticism. Both strands of Dennett’s criticism are then countered by analyzing the key components of Husserl’s method of phenomenological reflection: *epoché* and transcendental reduction, intentional analysis, eidetic variation, and intersubjective validation. Laying out the basic features of phenomenological reflection serves two purposes. First, it undermines Dennett’s methodological arguments, which are based on the unfounded assumptions that Husserl is committed to introspection, methodological solipsism, the first-person-plural presumption, and the lone-wolf approach. Second, it shows how Husserl’s own methodology can alleviate the more justified empirical worries concerning overinterpretation, underdescription, and disagreement. Finally, I argue that gradual weak skepticism is the only plausible form of phenomenological skepticism and conclude that Husserlian methodology is well-equipped to combat it.

## Introduction

There is a long tradition of questioning the viability and scientificity of reflective methodology. Back in the early nineteenth century, Auguste Comte stated in his *Cours de philosophie positive* (1830–1842):

“For all the two thousand years during which metaphysicians have thus cultivated psychology, they are not agreed about one intelligible and established proposition. […] ‘*Internal observation*’ gives almost as many divergent results as there are individuals who think they practice it.”^1^

To capture this still prevalent concern, Jean-Michel Roy has coined the term “phenomenological skepticism.” He defines phenomenological skepticism in terms of the current debate about the relevance of phenomenology, and first-person methods in general, in the context of cognitive science and the science of consciousness:

“[P]henomenological scepticism [is] the long standing objection that the traditional conception of phenomenology falls short of the basic requirements of science, because it cannot provide a knowledge endowed with a sufficient degree of reliability and objectivity.”^2^

It is no surprise that Roy focuses on Daniel C. Dennett as the most ardent present-day proponent of phenomenological skepticism. Much like other philosophical behaviorists before him, Dennett(2001/2018, p. 467) has voiced his distrust of first-person investigations, neither mincing his words nor lacking in rhetorical flair: “First-person science of consciousness is a discipline with no methods, no data, no results, no future, no promise. It will remain a fantasy.”

Such an unequivocal condemnation of first-person approaches has led some commentators to argue that Dennett has construed an overly simplistic picture of first-person methods in general and a straw man view of classical phenomenology in particular (see Zahavi, 2007; Cerbone, 2012). Conflating different ways of examining conscious experience from the first-person perspective, especially introspection and phenomenological reflection, has allegedly led Dennett to think that their flaws and limitations are similar, if not the same altogether. His undifferentiated view of first-person methods and shared mistrust of them can be seen as Dennett’s reason for rejecting all kinds of reflective endeavors, including Husserl’s phenomenology, as scientifically suspect.

Roy (2007) claims, however, that Dennett’s phenomenological skepticism should not be considered a wholesale dismissal of reflective efforts in general or even phenomenology in particular. On closer inspection, he maintains, Dennett employs a dual strategy of making use of some elements of Husserlian phenomenological methodology, while doing away with the rest^3^. On this reading, Dennett criticizes phenomenological reflection only on epistemological and methodological grounds, questioning its reliability and alleged lack of objectivity. At the same time, he is open to integrating Husserlian analyses into a naturalistic framework – in particular, into his “heterophenomenological” approach to the study of consciousness. This reading is supported by Dennett’s self-avowed “buffet approach” to Husserl^4^.

Those who regard Dennett as largely dismissive of phenomenology have also dissected his brand of skepticism. David Cerbone has reconstructed epistemological and ontological varieties of skepticism and offered detailed responses to ensuing questions concerning the accuracy and comparability of reflective knowledge. Dan Zahavi, in turn, takes on Dennett’s charge of methodological solipsism by highlighting that structures of experience are intersubjectively accessible objects of reflective investigation and that phenomenological analyses result in descriptions and arguments open to communal corrections. In answering Dennett’s critique, both Cerbone and Zahavi present Husserl’s phenomenology as a transcendental project that investigates the conditions of experience and the constitution of reality. From the transcendental perspective, it is possible to turn the tables on Dennett and question his commitments to the naturalization of consciousness and objectivistic scientific worldview^5^. Both Zahavi and Cerbone also argue that Dennett mistakes phenomenological reflection for (psychological) introspection and deem skepticism deriving from this equation misguided^6^. In contrast, Roy (2007) recognizes phenomenological skepticism as a pertinent problem – especially if one wishes to integrate Husserlian investigations into a naturalistic framework of cognitive science. In his mind, Husserl’s anti-naturalist *credo* and alleged commitment to infallibilism render “orthodox” Husserlian phenomenology vulnerable to Dennett’s objection.

I will draw from Zahavi’s and Cerbone’s arguments and develop some of them further in order to counter Dennett’s methodological criticism of Husserlian phenomenology. In taking on Dennett’s empirically oriented arguments as objections worthy of closer consideration, however, my strategy is closer to Roy and his integrative approach, although Zahavi’s and Cerbone’s reading of Husserl is more faithful to his work. It is true that Husserl saw all genuine skepticism (including doubting the epistemic value of reflection) as self-defeating and countersensical (*widersinnig*), since it implicitly assumes or makes use of what it explicitly denies (Husserl,1976, p. 174; cf. Husserl,1975, p. 120, 123). At the same time, he was well aware of the same kind of worries associated with casual reflection and psychological introspection that also motivate Dennett’s skepticism. Husserl(1976, p. 172) recognized that skeptical doubts concerning self-observation can be readily extended to all reflection, including phenomenological reflection. For this reason, I argue, Husserl’s phenomenological reflection is well-equipped to safeguard against them. In this respect, my conclusion also differs from Roy’s: rather than succumbing to phenomenological skepticism, Husserl’s reflective methodology offers tools for restraining our erroneous tendencies and mitigating the skeptical concerns.

I will begin by outlining Dennett’s empirically driven reasons for phenomenological skepticism (section “Dennett’s Empirical Arguments”). Then, I will reconstruct his methodological arguments against Husserlian reflective methodology (section “Dennett’s Methodological Arguments”). The focus will be on four methodological commitments Husserl allegedly makes: use of introspection, methodological solipsism, the first-person-plural presumption, and the lone-wolf approach. In section “Strong and Weak Skepticism,” I will take a closer look at the ensuing phenomenological skepticism by dividing it into strong skepticism, categorical weak skepticism, and gradual weak skepticism. In order to respond to phenomenological skepticism, I will explicate the basic elements of Husserl’s method of phenomenological reflection, namely *epoché* and transcendental reduction, intentional analysis, eidetic variation, and intersubjective validation (section “Phenomenological Reflection”). This has a dual function of dispelling Dennett’s methodological arguments while showing how Husserl’s methodology can alleviate the more justified empirical worries concerning overinterpretation, underdescription, and disagreement. Finally, I will conclude that gradual weak skepticism is the only plausible form of phenomenological skepticism and that Husserlian methodology is well-positioned to combat it.

## Dennett’s Empirical Arguments

Dennett’s critique of first-person methods can be divided into empirical arguments and principled methodological worries. First, it should be noted that Dennett neither categorically denies the possibility of reflective description of conscious experience nor questions that subjects have *some* privileged access to their own experience. The problem is, rather, that no refined technique providing reliable results and methodological guidelines for scientific use of first-person methods has been established. In other words, Dennett argues that subjective approaches to studying conscious experience have failed to meet the epistemological standards and methodological requirements of science. Phenomenology, in particular, has not met its goal of describing the contents of our conscious experience faithfully and reliably, without distortions or unfounded theorizing. By lacking a neutral method of description and a common ground for assessing its results, Dennett claims that phenomenology “has failed to find a single, settled method that everyone could agree upon” (Dennett, 1991, p. 44, 67–69). I will challenge Dennett’s assessment of methodological unanimity in section “Phenomenological Reflection.” Let us first break down the empirical and methodological reasons for his suspicion of first-person methods.

Dennett points out several restrictions on our capacity to reflect upon conscious experience. In arguing against the viability of first-person investigations, though, he uses empirical research unsystematically and sporadically at best. To support his case, Dennett also resorts to traditional philosophical arguments and utilizes illustrations from the history of philosophy and everyday psychology, supplemented with analogies, metaphors, and thought experiments. Nevertheless, I call the following arguments *empirical* because they either have some basis in actual empirical research or at least point to human psychological or cognitive tendencies and the limits of our reflective capacities that can in principle be empirically detected and tested in experimental settings^7^.

Dennett’s empirical arguments center around the claim that we are overconfident in our ability to ‘get it right’ when it comes to our own experience. This propensity comes to the fore in, at least, three forms: (1) underdescription, (2) disagreement, and (3) overinterpretation.

First, we seem to underestimate the blind spots of our reflective grasp of our conscious experience. Important features of ongoing experience go unnoticed and, in some cases, we seem to be demonstrably mistaken about them. Dennett’s favorite example is peripheral vision. According to Dennett, naïve reflection makes us think that our visual field is sharp and uniformly detailed not only from the center but also all the way to the boundaries. But even simple demonstrations (such as moving a playing card held at arms length from your side to the center of your visual field) show that, in fact, it is hard to identify objects in terms of their color or shape quite close to the center, even though you can detect movement. This so-called deficiency in our peripheral vision goes unnoticed because our eyes are normally continuously tracking and saccading in order to bring objects to the center of foveal vision. Instead of providing information in a manner of a “snapshot,” our visual field is much more undetermined and lacking in detailed visual content, with only a rapidly shifting clear center. This finding is said to surprise most people, even cognitive scientists, to the effect that in test settings many subjects confess being formerly mistaken about their visual field^8^.

Second, Dennett points out, in the Comtean vein, that factual disagreement and the lack of comprehensive data provide evidence for the *un*reliability of first-person methods. Dennett refers in passing to some examples from the history of empirical introspective psychology, like the unresolved debate about imageless thought (Dennett,1991, p. 59; cf. Roy,2007). His overall argument, however, does not rest solely on documented cases of disagreement. He also invokes the alleged lack of *positive* results of first-person investigations and the supposed inability to settle disputes if conflicts ensue. According to Dennett(1991, p. 44–45, 96; cf. Dennett, 1978, p. 185), phenomenologists, in particular, have failed to produce a catalog of all the items inhabiting our conscious experience, whose contents the experts could by and large agree upon. Instead of being a reliable communal activity of “pooling shared observations,” first-person investigations have allegedly lapsed into “the battle of ‘intuitions’,” where controversies are often met with “desk-thumbing cacophony” and “talking past everybody else” (Dennett, 1991, p. 66, 96).

Third, according to Dennett(1991, p. 67), this kind of “controversy and contradiction” – *contra* the sought-after mutual agreement – not only shows that our trust in high reliability of introspection is misguided; it also betrays the fact that we are prone to overinterpretation and unfounded theorizing about our experience. Dennett’s notorious example is the notion of the self, which he sees as a narrative creation. It may be a useful fiction, but the self nevertheless is something neither reliably found within our conscious experience nor verified by external observation (Dennett, 1991, p. 412; cf. Dennett, 1992). It is highly unlikely that a question as multifaceted as the nature and existence of the self could ever be settled in an empirical setting. Nevertheless, Dennett thinks the impasse of modern philosophy is telling: the chain of philosophers who all claim to be using a first-person method of some kind and assume that their “introspecting” could be readily replicated at will (Descartes, Hume, Locke, and their successors), have ended up in conflicting, and even opposing, views on whether there is a self at all and what its nature would be (see Dennett, 1991, p. 66–67, 412–413). The variety of opinions indicates that humans have a tendency to fabulate descriptions of their own experience – to “fill in the gaps, guess, speculate, mistake theorizing for observing” (Dennett, 1991, p. 94)^9^.

More recently, Eric Schwitzgebel has suggested three types of argument, all based on empirical case studies and their philosophical analysis, as to why introspection is prone to error: (1) There seems to be more variation in people’s introspective reports than is plausible to assume there are underlying differences in ways we experience things (argument from variation)^10^. (2) There are cases where most people are clearly and crudely mistaken about quite basic features of their own experience (argument from error)^11^. (3) Sometimes introspection yields remarkably inconclusive results (argument from uncertainty)^12^. One can notice the overlap with Dennett’s arguments; in fact, Dennett can be seen to apply these argumentative strategies in undermining our confidence in the reliability of reflective investigations.

I will return to the empirical arguments when assessing Dennett’s phenomenological skepticism in section “Strong and Weak Skepticism.” It is, however, already important to note that the restrictions detailed above are not specific to phenomenological reflection *per se*. The implicit argument found in Dennett is, rather, that phenomenology suffers from the same kinds of empirical limitations and misgivings as everyday reflection and earlier introspective psychology, until proven otherwise. In Dennett’s view, we are bound to overstep, or to ignore altogether, the limits of our reflective cognitive capacities. In section “Phenomenological Reflection,” I will formulate a Husserlian response to the challenges posed by Dennett’s empirical arguments by showing how phenomenological reflection actually safeguards against the perceived problematic tendencies of casual reflection and introspection and offers methodological tools for alleviating overinterpretation, underdescription, and disagreement.

## Dennett’s Methodological Arguments

Empirical arguments in the wide sense outlined above can be separated from *methodological* arguments, which are based on Dennett’s general view on what counts as science and what scientific methodologies allegedly permit. Dennett’s methodological critique of phenomenological reflection is based on the distinction between first-person and third-person methods and their respective data^13^. In *Consciousness Explained*, Dennett makes a categorical claim that scientific theories can only be constructed from the third-person perspective. For Dennett, this means using objective methodologies that rely only on data that is intersubjectively accessible and verifiable, i.e., available for external observation and open for independent validation. In order to be considered as scientific, first-person investigations should also be able to constitute a reliable communal practice based on shared observations. But instead of delivering identical results or even findings that *could* be replicated, first-person investigations arguably end up relying on private access to subjective conscious experience and produce indefeasible statements (see Dennett, 1991, p. 66, 70–71, 2003, 2007; see also Overgaard et al., 2008).

Dennett sees his methodological criticism as resting on a standard conception of science and does not admit any need to reform or even adjust it to accommodate first-person methods. Therefore, he builds his case by pointing out *deviations* from the presumably widely accepted standards of natural sciences, rather than specifying criteria for scientific objectivity or reliability more explicitly^14^. By way of approximating these criteria, first-person methods fail to meet at least three underlying requirements of science: (1) they depend on a single observer and use private data as evidence (rather than producing intersubjectively testable statements on publicly available objects of investigation); (2) they rely on unreliable introspective practices (rather than reliable observation producing mostly accurate and corrigible results); (3) they provoke disputes about the methods and disagreement about their results (rather than producing unanimity and ways of settling the disputes). Let us call these criteria intersubjectivity or publicity, reliability, and agreement^15^.

What reasons does Dennett give for his methodological concerns? In addition to citing reflective errors and empirical restrictions examined in the previous section, he turns to the history of psychology for indirect support of his position. Dennett sees the decline of introspective psychology and the resulting advancement of behaviorism in the first half of the twentieth century as bearing witness to the insurmountable problems of first-person methodology in general. In response to introspective psychology’s inability to compare, replicate, and validate its results, behaviorists accepted only intersubjectively verifiable methods and dismissed any purported facts about mental events as data: since one cannot “look directly into” the minds of others, one should stick strictly to observation “from the outside.” What is central to Dennett’s argument is that the ensuing methodological shift was not restricted to the behaviorist school or confined to a certain period. Rather, in *Consciousness Explained*, he argues that suspicion of first-person methods has since become the guiding norm of *all* research in experimental psychology and neuroscience^16^. Dennett then proceeds to insist that a theory of consciousness *must* be constructed from the third-person perspective, “using the data that scientific method permits” and “never abandoning the methodological scruples of science” (Dennett, 1991, p. 71–72).

The methodological critique is also aimed at phenomenology more directly. By adopting what Dennett takes to be the standard first-person perspective in writing about consciousness, phenomenologists allegedly buy into what he calls the “first-person-plural presumption.” By the *first-person-plural perspective* Dennett alludes to the resulting mode of investigation or a style of philosophizing where one describes first-hand in a monolog what is given in *my* conscious experience *and* assumes that others will agree. While Descartes’ meditations and the first-person accounts favored by the British Empiricists are Dennett’s paradigmatic historical examples of the approach, he sees modern day phenomenologists proceeding in a similar fashion. The way I read Dennett’s criticism is that adopting the first-person-plural perspective leads to methodologically dubious practices in at least two related ways. First, it promotes problematic, and even careless, ways of generalizing from *my own* singular experience. Second, it paints a simplistic, and even erroneous, picture of first-person investigations as an agreement-producing practice, in which first-person accounts are readily reproduced by *others* by making personal inner observations and arriving at the same results (Dennett, 1991, p. 66–67, 70).

Dennett does not claim that first-person investigations are useless, not to even mention impossible. He admits that they may very well offer motivation, illustrations, and even guidance for scientific theories. But in his mind, they do not yet provide the kind of reliable data needed for the science of consciousness. First-person investigations become scientific only after the private reflective findings are turned into intersubjectively accessible third-person data by conducting controlled experiments with naïve test subjects. Dennett sees classical phenomenology committing itself to a “lone-wolf” approach, where both the subject and the object of investigation are one and the same person. By Dennett’s standards, this puts phenomenologists in a similar position with experimenters who would run pilot studies on themselves but fail to confirm their findings with other test subjects. What Dennett seems to imply is that lone-wolf phenomenologists do not even try to meet the obligation of testing their insights intersubjectively and in interaction with others. Instead, they rely on “personal introspection” as the only evidence needed for substantiating claims about conscious experience. For Dennett, it is widely accepted that no defensible first-person *science* can be built on these grounds (Dennett, 2003).

In addition to the first-person-plural presumption and the lone-wolf approach, Dennett sees Husserlian phenomenology as married to a third methodological commitment, namely methodological solipsism. Dennett’s methodological argument is ambiguous on this score. On the one hand, by methodological solipsism he means adopting *a research strategy* in which the experiencing subject is investigated in isolation from their environment, that is, not historically, linguistically, or causally embedded in the world^17^. On the other hand, it concerns different ways of *implementing* this strategy, that is, the methods used in gaining access to and studying the proper domain of investigation dictated by methodological solipsism. For the time being, it is sufficient to say that Dennett seems to think that an introspective approach combined with a methodological procedure called *epoché* or “bracketing” leads Husserl to exclude the outer world and its real objects from phenomenological reflection and to restrict its scope to the internal or mental domain and the subjective world of a person.

Supplementing this threefold negative critique, Dennett offers a more positive vision on integrating certain kinds of phenomenological investigation into a naturalistic framework. Instead of examining conscious experiences directly as they appear to a single subject living through them, Dennett suggests that the science of consciousness should study (other) people’s *beliefs* about their experiences and their *reports* expressing them in experimental setting. He famously labels this methodological change of focus as a transition from *auto*phenomenology (phenomenology of oneself) to *hetero*phenomenology (phenomenology of the other). The researcher should not introspect, describe, and “catalog” conscious experiences as the sole reflector. Instead, she should gather first-person perspective reports of what people think of their experiences and interpret them by adopting the intentional stance^18^. Thus, what becomes “the data” is not the experiences themselves but people’s beliefs *about* them, expressed in verbal form or through behavioral manifestations, like pushing a button or reacting in certain ways in different circumstances: “the reports *are* the *data*, they are not reports *of* the data” (Dennett,1993; see also Dennett,2003). Dennett(1991, p. 72) presents heterophenomenology as a neutral method of capturing even “the most private and ineffable” experiences without leaving the framework of objective third-person science and its standard methodologies. The picture of an armchair philosopher meditating in solitude is replaced with a figure of an empirical researcher gathering factual data and interpreting it with anthropological, psychological, and narrative insight.

## Strong and Weak Skepticism

Dennett’s phenomenological skepticism is based on the empirical and methodological arguments outlined above. At the outset, Dennett does not espouse strong skepticism, which claims that reflection has no access to anything real at all. He does not wish to prejudge the issue by simply denying the existence of conscious experience (or mental states) or identifying them with something else, e.g., information-bearing brain events^19^. Instead, Dennett’s aim is to create a neutral method of describing experiences that is not committed to any ontological theses on the existence of experiences. This is why he presents his heterophenomenology as a method that investigates conscious experience indirectly *via* interpreting people’s reports. In this way, Dennett hopes to take seriously how things *seem* or *appear* to subjects (i.e., what it is like to them), but grant them neither infallibility nor a final say on the fact of the matter (i.e., what is going on in them). This would arguably help to constrain unfounded theorizing about the nature and metaphysical status of objects of investigation and their assumed causes. For Dennett, commitment to neutrality is needed not only to secure a reliable way of gathering data, that is, for mapping out and describing experiences carefully, accurately, and comprehensively; a neutral way of extracting a “heterophenomenological catalog” also provides shared ground for settling reflective disputes, in order to avoid “the battle of ‘intuitions”’ allegedly characteristic of first-person investigations (Dennett, 1991, p. 96).

Dennett’s plea for ontological neutrality comes with what he calls “metaphysical minimalism” (Dennett, 1991, p. 95). According to this principle, the objects of heterophenomenological investigation should not be taken at face value and be accepted as real, but taken only as assumptions, “theorists’ fictions.” Dennett leaves it to the empirical sciences to decide whether items described by heterophenomenology correspond to anything real or not, that is, whether they exist as real objects (as brain states, mental states, cognitive processes or the like) (Dennett, 1991, p. 81, 96, 98). Zahavi (2007) has claimed that here Dennett is actually an eliminativist in disguise, since his “metaphysical minimalism,” in fact, leads to the denial of existence of experiences (cf. Cerbone, 2003, p. 133). Carr (1998) has pointed out problems with the fiction analog, starting with the fact that the metaphor runs counter to commitment to ontological neutrality, since *fiction* is not a metaphysically neutral term. In some cases, Dennett also seems to downright deny the actuality of experiences and in other cases he calls experiences “theoretical constructs” (see e.g., Dennett, 1991, p. 95, 157, 365). When Dennett(1991, p. 83) states that for a heterophenomenologist it makes no difference if her test subjects are “liars, zombies, or parrots dressed up in people suits,” his ontological neutrality seems to lapse into what Cerbone(2012, p. 14) calls ontological skepticism: “it does not matter all that much whether anything really does correspond – whether there is a ‘fact of the matter’ about experience – since the beliefs are all that we have to express, report”. More recently, Dennett has openly endorsed a strong anti-realist position called illusionism. Illusionism claims that phenomenal consciousness only *seems* to exist but is in fact illusory and that phenomenal properties are not real in the sense of being instantiated in the world (Frankish, 2016). For Dennett (2016), illusionism is not only the correct interpretation of his own account, but also the *default* view everyone engaging in scientific study of consciousness should hold, until proven otherwise. It is hard not to interpret these commitments as strong skepticism, well beyond the coveted ontologically neutral standpoint of heterophenomenology.

Dennett’s general motivation for embracing such deflationist views can be traced back to his philosophical behaviorism, naturalistic approach to consciousness, and commitment to the third-person conception of standard science. Zawidzki has suggested that Dennett’s strategy is to deflate *and* revise our manifest concepts of intentionality and consciousness in terms of publicly observable patterns of behavior. As intersubjectively verifiable phenomena, describable from the intentional stance and captured by heterophenomenology, they are, then, arguably easier to reconcile with the scientific image, i.e., what standard science accepts and rules out (Zawidzki, 2007, p. 154–156; cf. Dennett, 1987, p. 25). A case in point is Dennett’s adamant rejection of *qualia* or phenomenal properties conceived as ineffable, intrinsic, private, systematically incomparable, and directly apprehensible features of conscious experience (Dennett, 1988; Frankish, 2016; cf. Dennett, 2020). Dennett denies the existence of *qualia* so defined as objectively undetectable, intersubjectively untestable, and, thus, incompatible with standard science. Hence, Dennett regards investigating consciousness scientifically in *these* terms impossible (see Zawidzki, 2007, p. 167–169). Whether Dennett’s self-avowed eliminative materialism and verificationism about *qualia* amounts to denying conscious experience conceived in less contentious terms is debatable^20^. Whatever the case, Dennett has recently admitted that his battle against *qualia* as real properties of experience has made him overlook tensions in his discussion of conscious experience in terms of *seeming(s)^21^*. His ongoing polemics against “qualophiles” might, therefore, explain not only rhetorical excesses but also perceived ontological and epistemological inconsistencies in Dennett’s views^22^.

Perhaps a combination of deflationist-revisionist strategy and unyielding resistance to *qualia*, then, accounts for Dennett’s apparent commitment to strong skepticism. In the next section, I will argue, however, that Dennett can defend his maxim of ontological neutrality and do away with *qualia* without espousing ontological skepticism, let alone illusionism or eliminativism about conscious experience. Therefore, I will focus on the weaker and more plausible forms of skepticism.

Weaker forms of skepticism only deny the possibility of (scientific) knowledge about our conscious experience. Dennett’s epistemologically motivated skepticism rests on a conviction that we are far less immune to error than we think, a view that is supported by the types of empirical arguments outlined above. Dennett (2001/2018) refers specifically to two kinds of false beliefs, in which our assertions about our conscious experience fail to overlap with the fact of the matter: (i) false positives and (ii) false negatives. By *false positives* he means cases in which we believe we are conscious of more than what is in fact going on in us (as Dennett pointed out with the example of unsuspected deficiency in our peripheral vision). Conversely, by *false negatives* he refers to cases in which we do *not* believe we are conscious of things that are or were actually going on in us (as is demonstrated, for instance, by psychological experiments using masked priming, showing influence undetected from the first-person perspective). It is safe to assume that Dennett sees our inability to access all the workings of our own mind *via* “inner observation” as leading to both overstepping what can be reliably said about our experience (argument from overinterpretation) and overlooking important features of our experience (argument from underdescription) (see Dennett, 1991, p. 68–70, 94). He explicitly states false negatives and false positives as reasons for his skepticism about the truthfulness of first-person reports and as informing his insistence on bracketing the veracity of subjects’ beliefs (methodological argument for ontological neutrality) (Dennett, 2001/2018, p. 457–458; cf. Dennett, 1991, p. 94).

It should be noted, however, that issues of false positives and false negatives reveal more about the restricted scope of reflection and uncertainty of its results than about the impossibility of first-person knowledge in general. It does not follow from the fact that some factors or features of our experience are not directly accessible from the first-person perspective that *all* of consciousness is inaccessible. Similarly, the inaccuracy of some reports does not merit the conclusion that conscious experience cannot be faithfully described at all. After all, one does not have to treat all reflective descriptions as equally reliable or assume that our experience is fully transparent to us (Goldman,1997, p. 529, 532; Roy, 2007, p. 14). The problem is not that we can know *nothing* about our experience, but that we are overstating what we know (cf. Nisbett and Wilson, 1977). Another way to put it is to say that we take too large a portion of our mental and cognitive processes to be available reflectively and think that conscious experience is more transparent than it really is.

Following Hurlburt and Schwitzgebel(2007, p. 27), it might be helpful to distinguish skepticism about first-person access to non-conscious processes (such as cognitive mechanisms, but also psychological traits, behavioral dispositions, etc.) from skepticism about the self-reports of conscious experience. The defender of reflective knowledge can readily admit that the limits of privileged access are not always clear and that we might occasionally “overstep” even the “self-imposed restraints” (Dennett, 1991, p. 68) to stick within those limits. Fallibilism in these regards does not merit general skepticism about *all* possible reflective knowledge. I call this the *gradual version of weak skepticism*. As I will argue in the next section, Husserl’s phenomenological reflection is well-positioned to take these worries into account.

Dennett’s skeptical argument against phenomenology, though, also comes in a more categorical form. Dennett(1991, p. 67) claims that the Cartesian tradition sees us as either infallible (i.e., always guaranteed to be right) or at least incorrigible (i.e., no one can correct us) when it comes to knowing our conscious experience *via* self-observation. He also speaks of a “long-standing philosophical tradition” guilty of assuming that “*we all agree* on what we find when we ‘look inside’ at our own phenomenology” (Dennett, 1991, p. 66). Here, Dennett seems to imply that some kind of immunity from error is inherent in the aforementioned first-person-plural presumption. As an extension of the principle of charity, Dennett does propose granting limited incorrigibility to test subjects in heterophenomenological settings. People might be unreliable guides in what is going on in them, but they should be taken at their word on *what it is like* to be them or how it *seems* for them. But if one, then, goes on to extrapolate from one’s own particular experience or engages in theorizing about the nature of conscious experience, this privilege is renounced and the person reflecting must be treated as a fallible theorizer (Dennett, 2002). Following this view, appealing to unchallengeable first-person authority or “papal infallibility” (as Dennett likes to put it) in an attempt to secure reflective knowledge about the general features of conscious experience is both methodologically unwarranted and empirically suspect (if the empirical arguments are any indication of our liability to error). Discrediting reflective knowledge *in toto* by invoking the unfounded reliance on infallibility or incorrigibility extended beyond its methodological confines can be named the *categorical version of weak skepticism*.

It is important to note that Dennett’s categorical skepticism does not hinge on the claim that autophenomenology always or even typically gets it wrong. The charge is, rather, that autophenomenology claims first-person authority, while not acknowledging that reflection is always *susceptible* to error (see Dennett, 2002, 2007). It is not perfectly clear whether Dennett sees Husserl as being guilty of this traditional tenet of infallibility, but this interpretation merits a closer look. In the debate about skepticism, commentators like Roy see Husserl as defending the indubitability of phenomenological reflection in general: “Husserl was a firm believer that the immediate knowledge at the source of the phenomenological inquiry, which he technically labeled immanent intuition, is immune to error” (Roy, 2007, p. 6).

One could also claim that Husserl did not see the scope of reflection as limited. At least in principle, Husserl argues that we can, at any time, turn our attentive regard to our ongoing experience and to what is “straighforwardly” experienced in it. In other words, whenever there is conscious experience, we are free to reflect upon it, thus creating a second-degree act of reflection, which takes the previously lived-through experience and its meaning-content as its object (see Husserl, 1950, §§14–15, 1976, §§77–78). One possible reading of the claim is that the potential range of reflection is equivalent to the domain of experience as a whole. Perhaps this is what led Roy(2007, p. 14) to argue that Husserl was committed to a “double thesis,” according to which “the whole of the mental is manifested to us, and that it is manifested to us as it really is,” namely without distortions or limitations.

If Husserl admits neither restrictions to the scope of reflection nor uncertainty of its results, does he not succumb to the kind of infallibility and incorrigibility Dennett’s categorical weak skepticism argues against? In order to answer this question in the negative, let us next turn to the methodological elements of phenomenological reflection.

## Phenomenological Reflection

In this section, I will reconstruct the key features of Husserl’s method of phenomenological reflection. The reflective methodology can be articulated by dividing it into four elements: (1) *epoché* and transcendental reduction, (2) intentional analysis, (3) eidetic variation, and (4) intersubjective validation.

This reconstruction has both a negative and a positive function. First, it shows that Husserl’s reflective methodology is not committed to the kind of presumptions Dennett claims, while addressing the more justified skeptical worries and potential pitfalls associated with first-person methods. Second, it outlines phenomenological reflection as a systematic method in its own right. Contemporary efforts to develop Husserlian approaches to conscious experience and to integrate them with cognitive sciences have yielded numerous applications, including direct and indirect use of phenomenology in experimental settings^23^. Whether one employs Husserlian methodology as a toolbox for compound methods (Schmicking, 2010) or wishes to develop it further as a self-standing method, understanding the core features of phenomenological reflection is invaluable. Hence, the methodological and epistemological issues discussed in this section have further relevance to the phenomenologically informed cognitive science and the interdisciplinary study of consciousness.

### *Epoché* and Transcendental Reduction

Already in *Logische Untersuchungen* (1900–1901), Husserl called “presuppositionlessness” a basic principle of phenomenological research. This *Voraussetzungslosigkeit* demands refraining from metaphysical presumptions, psychological presuppositions, and theories and explanations of other sciences (both empirical-inductive and axiomatic-deductive), in order to consider experiences faithfully (Husserl, 1984, p. 24–28). Furthermore, phenomenological reflection has to fight both habits and tendencies rooted in our psychological development and linguistic problems in describing experience and communicating the results (Husserl, 1984, p. 14–15). In other words, from early on, Husserl points out several potential sources of distortion and bias in reflection and description, professing a need to constrain them methodologically.

Throughout his career, Husserl also carefully heeded how reflection modifies its object, i.e., lived experience. In fact, reflecting, as Husserl sees it, is far from a process of simply recording and reporting what is already there – a matter of merely “looking and seeing” as Dennett(1991, p. 55, 66) claims we tend to think. At first, we are confronted by “dumb” or “mute” (*stumm*) experience (Husserl, 1950, p. 77). The cognitive value of phenomenological reflection is in its power to modify the previously unregarded and straightforward pre-reflective experience, in order to thematize and articulate it^24^. Dennett *could* argue that it is precisely this modification that alters and potentially distorts experience, making its faithful description impossible and opening up unconstrained theorizing^25^. Indeed, Husserl would admit that it is impossible to repeat or replicate the original experience exactly as it was lived through. At the same time, he would stress that this is not the goal of phenomenological reflection in the first place. After all, when we are immersed in our everyday living and take interest in the environing world, our experience is often seemingly undifferentiated. It is the reflective attitude that makes it possible to uncover, analyze, describe, and even clarify experiences in terms of their constituents and basic structure. To be sure, Husserl is well aware of the risks of reflective modification leading to “metaphysical construction” (Husserl, 1950, p. 177) and “reading into [experiences] more than is purely seen” (Husserl, 1950, p. 74) that Dennett warns us about. It is precisely for this reason that Husserl(1950, p. 74) sees preserving the “unprejudicedness” (*Vorurteilslosigkeit*) of descriptions as essential for acquiring (critical) reflective knowledge.

How can we combat the aforementioned difficulties methodologically and pursue the epistemological potential of reflection without prejudice? It was not until *Ideen I* (1913) that Husserl supplemented his basic principles of phenomenological investigation with a systematically spelled out doctrine of the phenomenological reduction(s). At its core, the doctrine involves an element of abstaining from judgments and thus “bracketing” or neutralizing our prior commitments; this is what Husserl famously calls *epoché*. Husserl speaks of “bracketings” (as well as reductions) in plural and alludes to different steps of the process, indicating that the scope of the procedure in question can be gradually expanded and restricted. Husserl’s motive for this mode of presentation is both didactical and critical: we need to be constantly reminded not to let premises from other sciences (not only natural sciences, psychology, and *Geisteswissenschaften* like history and cultural and social sciences, but also formal-eidetic sciences such as pure logic and mathematics) carry over to phenomenology and instructed not to make use of their results as readymade stocks of knowledge. In his mind, presenting the needed “bracketings” step-by-step protects methodologically against common misconceptions (both contemporary and historical) and prepares for the avoidance of the constant threat of categorical mistakes and other ingrained habits of thought (see Husserl, 1976, §1, §56, §59, §61).

In the pregnant sense, however, *epoché* amounts to more than a series of exclusions. Rather than suspending our beliefs one by one or domain after domain, the ultimate aim is to “put out of action” *all* positing that characterizes our so-called natural attitude and its “general thesis.” In other words, *epoché* has a universal goal of putting our everyday doxic attitude toward the world on hold and bracketing the related ontological commitments concerning its objects (Husserl, 1976, §§30–32).

Dennett does recognize Husserl’s *epoché* as a possible way forward for securing the neutrality of descriptions. He even presumes heterophenomenology applies a third-person analog to *epoché* in reserving the judgment about the veracity of subjects’ beliefs and seeking theory-free descriptions (Dennett, 2003). Even though the aim of neutrality might be the same, Husserl’s and Dennett’s varieties of “bracketing” are, in practice, quite different. In fact, as Cerbone(2003, p. 111) has noted, their views are almost mirror images in this respect: whereas Husserl brackets the whole of reality *including* ourselves as part of the world, Dennett puts into brackets only the reality of consciousness. Dennett, thus, comes close to doubting (ontological skepticism) or even denying (illusionism, eliminativism) the existence of conscious experience without questioning his own commitment to ontological naturalism. Husserl(1976, §§31–32, §109) himself takes pains to ensure that *epoché* should *not* be understood as a methodological *doubt* of existence, let alone as *negating* the actuality of conscious experience and the world. The aim is rather “neutralizing” or “parenthesizing” our ontological and theoretical commitments in order to examine them.

Following Husserl’s original idea of *epoché* more closely would arguably help Dennett to attain his goal of ontological neutrality, without lapsing into strong skepticism. Positing superfluous entities, such as *qualia*, as a stand-in for phenomenal properties of experience could be avoided while resisting the opposite pull of simply reducing or identifying conscious experience with something else (see Dennett, 1969/1986, p. 112–113, 1991, p. 459–460). In this way, one could salvage ontological neutrality as one of the main motivations for both classical and heterophenomenology by separating it from the more problematic principle of metaphysical minimalism. Furthermore, a more faithful understanding of *epoché* could relieve some of the concerns behind gradual versions of weak skepticism. As a preparatory stage of Husserl’s reflective methodology, the negative aim of *epoché* is to secure the aforementioned “unprejudicedness” of descriptions by identifying and avoiding different sources of bias. At the very least, *epoché* would safeguard against the sort of “impromptu theorizing” and over-interpretation Dennett is worried about. This is only half of the story, but Dennett could take this line in order to incorporate the negative function of *epoché* into heterophenomenology instead of interpreting *epoché* as a form of methodological solipsism.

The other option is to argue that Dennett cannot achieve ontological neutrality at all without also reflecting upon the presuppositions and the metaphysical baggage of his own naturalistic commitments (see Cerbone, 2012). In fact, it is arguably paramount for any theory of consciousness to reflect upon its basic assumptions concerning the place and function of consciousness in what is taken to be objective reality^26^. In addition to the negative move of excluding prejudices, *epoché* enables a positive methodological step or a change of attitude that Husserl calls *transcendental reduction*. According to Husserl(1950, §15), this philosophical procedure enables a new kind of *transcendental* reflection since it focuses on the constitution of reality and its preconditions in conscious experience. Husserl contrasts transcendental reflection with natural or psychological reflection which takes its objects to be part of the mental or “inner” domain as opposed to physical reality or the outer world. Transcendental reflection opens up philosophical inquiry into the (inter)subjective sources of meaning-formation or sense bestowing, constituting our understanding of and our belief in what is real. In sum, a fully effected *epoché* conjoins the positive and negative aspects of the method by *opening* a field of (transcendental-)phenomenological research and *securing* it from intrusive influences (Husserl, 1976, §32, §61).

The transcendental argument against Dennett, and all naturalistic positions for that matter, maintains that explaining the relationship between consciousness and reality without reflecting upon how conscious experience shapes our sense of reality in the first place is question-begging (Zahavi, 2007, 2017, p. 144ff; Cerbone, 2012). Along these lines, it can be claimed that Dennett fails to appreciate the fundamental methodological role of *epoché* and the ensuing transcendental reduction. Rendering *epoché* simply a matter of reserving judgment, suspending presuppositions, and striving for a theory-neutral description of experience (see Dennett, 2003) might partly capture the negative function of bracketing. However, as Zahavi has argued, refraining from preconceptions, speculation, and causal explanation in favor of unprejudiced description is neither the novel feature of *epoché* nor its ultimate goal. Presuppositionlessness was already one of Husserl’s core principles before his transcendental turn and the introduction of *epoché* as an explicit method. Focusing only on what is excluded would miss the positive side of universal *epoché* as bringing about a thorough change of attitude toward the whole of reality (Zahavi, 2002, p. 111–112, 2019).

However, Dennett *does* think that *époche*, as Husserl applies it, actually involves a methodological change in how the subject’s relationship to their environment is to be analyzed. This is, after all, implied by his methodological argument that (auto)phenomenology is committed to methodological solipsism and the lone-wolf approach. According to Dennett’s interpretation of Husserlian methodology, *epoché* serves as the way of implementing methodological solipsism by bracketing the outer or real world. Thus, it circumscribes the field of study and limits investigation to inner observation of a single subject. Consequently, phenomenological reduction amounts to nothing more than a dubious “introspectionist bit of mental gymnastics” leading to one’s *own* notional world (Dennett, 1987, p. 153, 157–158, 161). In other words, Dennett does not settle for his more modest claim that the goal of *epoché* is neutrality in the general sense of putting our presuppositions on hold when describing experience. Instead, he adopts a more problematic line of (mis)interpretation by holding that *epoché* and the ensuing reduction are needed for reorienting our focus from worldly objects to one’s own inner experience^27^.

It seems fair to conclude that Dennett’s reading of *epoché* (and reduction) is at best selective and partial. At worst, it distorts his overall conception of phenomenological reflection, especially regarding its relation to the world and to the single reflecting subject. It is not uncommon to treat *epoché* and reduction as an inward turn leading into a solitary individual consciousness; after all, this fuels the interpretation of Husserl as a methodological solipsist (see e.g., Varela et al., 1991/2016, p. 16–17). In the next subsections, I will show, however, that seeing reflection as a solipsistic and introspectionist technique leads Dennett to overlook other important aspects of Husserlian reflective methodology. Turning to Husserl’s ideas of intentional analysis and eidetic variation not only demonstrates why Dennett’s interpretation of Husserl as a methodological solipsist is wrongheaded; Dennett’s failure to acknowledge these ineliminable features of Husserl’s methodology also makes clear how his misconstrual of *epoché* in the end contributes to his claim that Husserlian phenomenology is a form of lone-wolf autophenomenology operating in the first-person-plural presumption.

### Intentional Analysis

In weighing Dennett’s case against Husserl, perhaps the most revealing aspect is what is missing from his charge of methodological solipsism. To my knowledge, Dennett does not discuss Husserl’s analysis of intentionality in any detail, save for a passing reference to *hyle* (Dennett, 1978, p. 184, 333) and comments on *noemata* as “the pure objects of conscious experience” (Dennett, 1991, p. 44) or “intentional objects constituted on a personal level” (Dennett, 2007, p. 259). As far as I can see, the nearest he gets to closing in on Husserl’s account of intentionality is in his allusions to Brentano’s doctrine of intentionality. In fact, Dennett’s charge of methodological solipsism can be dissected into mentalistic, internalist, and fictionalist components, and there are echoes of Brentano’s doctrine in each of these characterizations. Therefore, it is worth briefly tracing the Brentanian elements in Dennett’s reading, before presenting Husserl’s own version of intentional analysis as an answer to both Brentano’s position and Dennett’s charge of methodological solipsism^28^.

The mentalistic aspect of methodological solipsism is encapsulated in Dennett’s claim that *epoché* brings “the essence of the mental” to view by “bracketing the real world” (Dennett, 1987, p. 161). Dennett does not elaborate on what he thinks is revealed by this, but one can safely assume that intentionality as an essential structure of conscious experiences is amongst those features. The idea of intentionality as an intrinsic and exclusive feature of *mental* phenomena has its origins in Franz Brentano’s work. This is acknowledged by Dennett(1987, p. 67) who speaks of Brentano’s Thesis as characterizing intentionality as “the mark of the mental” and the defining feature of mental phenomena: “all and only mental phenomena exhibit intentionality” (Dennett and Haugeland, 1987)^29^. The mentalistic side of methodological solipsism blends with internalism in Dennett’s discussion of intentional *objects*. Dennett(1987, p. 153, 161) holds that “the real referents” of our beliefs are more or less inaccessible to introspective autophenomenology of Husserl and Brentano. If shared reality and its real objects are effectively pushed outside the scope of reflective investigations, how can phenomenology account for what is *intended* in conscious experiences? Here, Dennett introduces the fictional element to his reading of methodological solipsism. He proposes that one can make sense of what subject’s experiences are *about* by positing a *fictional* world consisting of whatever objects, events, etc. the subject happens to believe in. This notional world can then be described by taking those items as the notional referents of subject’s internal representations (Dennett, 1987, p. 118, 153, 155, 158–159, 1991, p. 81). It is in this move from internalism to fictonalism where Brentano’s influence is at its strongest. Dennett(1987, p. 153) more or less identifies what he calls notional objects with Brentano’s intentional objects. Furthermore, he alludes to Brentano’s account of intentional inexistence by acknowledging his merits in discussing the “relation*like* status” of mental phenomena with *non-existent* intentional objects (Dennett, 1987, p. 159) and guaranteeing an odd sort of existence to intentional objects as logical constructs (Dennett, 1978, p. 181).

Challenging the mentalistic, internalist, and representationalist readings of Husserl’s account of intentionality (e.g., McIntyre, 1988) in general is beyond the scope of this article^30^. Suffice to say that already in his early philosophy culminating in *Logische Untersuchungen*, Husserl was critical of similar features in Brentano’s account^31^. First, Brentano’s claim that an intentional relation demarcates psychical phenomena from physical phenomena can be challenged as mentalistic. Not only does it presuppose that all experiences considered “psychical” would take the form of an act directed at an immanent or mental object, but it also excludes phenomena like perceptual sensations (*Empfindungen*) from the mental domain as something “physical” (or, perhaps, physiological) (Husserl, 1984, p. 377–383, 406–407). Second, and relatedly, Brentano’s representationalism (i.e., the claim that all psychical phenomena are either representations or founded upon representations) leads him to overlook the non-intentional aspects of experience, such as sensory feelings (e.g., pain and pleasure) and bodily sensations (tactual, visual, olfactory, etc.) (Husserl, 1984, p. 382–383, 406–410). Classifying and determining phenomena in this way delimits conscious experience as a whole, restricting it to one of its subclasses, namely representational psychical acts directed at objects. Thus, Brentano ends up circumscribing the field of descriptive psychology more narrowly than Husserl delineates phenomenological investigations. Husserl’s terminological remarks regarding Brentano’s expressions pose a further challenge to mentalistic and representationalist readings of intentionality. In short, in Husserl’s mind one should avoid speaking of “mental objects,” “immanent objectivities” and “mental inexistence,” as if the objects intended were intramental parts of experience, enclosed or contained within consciousness (Husserl, 1984, 383–388). Third, Brentano’s internalism rests upon an epistemologically and metaphysically unfounded distinction between inner perception and outer perception. This dichotomy leads Brentano to grant inner perception the kind of immediacy and infallibility Dennett ascribes to autophenomenology and to prioritize it over fallible outer perception. Furthermore, the distinction is not metaphysically neutral, since it presupposes an outside world standing against an inner domain of consciousness. By the same token, Husserl claims that psychologists often draw a false antithesis between introspection and outer perception (see Husserl, 1984, p. 13).

More importantly, Dennett does not acknowledge that later in his career Husserl criticized Brentano precisely for the lack of what Husserl calls *intentional analysis* (see Husserl, 1950, p. 86, 1952, p. 59, 1954, p. 237, 1974, p. 252). Husserl maintained that intentional experiences can and should be described reflectively in terms of their non-independent structural features or moments and their correlative intentional objects. For Husserl, intentionality is not mere *aboutness*, as Dennett and others define it (Dennett and Haugeland, 1987). It is not merely a formal feature of directedness of consciousness, namely the fact *that* conscious experience is consciousness *of* something. On the contrary, intentional relation in the pregnant sense includes also *how* and *of what* we are conscious of in different kinds of experience (Husserl, 1976, p. 74; cf. Husserl, 1950, p. 71–72).

In *Ideen I*, Husserl(1976, p. 349) promoted reflecting conscious experience in terms of its hyletic, noetic, and noematic elements. On the one hand, phenomenological reflection may focus on the subjective side of experience by engaging in *noetic* analyses of different modes or types of consciousness (such as perception, imagination, and recollection) that give form and sense to experiences and their modal differences (such as clarity and distinctness). The subjective side also includes the so-called *hyletic* components providing material and content to experiences: the aforementioned perceptual sensations and sensory feelings, but also other types of sensual and bodily experiences related to affect, volition, desire, and sensibility, such as impulses, urges and drives, and even aesthetic pleasure. As non-independent moments of experience, they are not in themselves intentional but they function as “bearers” or “carriers” (*Träger*) of intentionality, and, as such, they partake in establishing and sustaining “consciousness of something” (Husserl, 1976, p. 74–75, 192–194, 1984, p. 409–410). On the other hand, one may attend to the objective side of experience in *noematic* analyses. These investigations focus on how intentional objects in different categories (such as a perceived tree, an imagined centaur, and a remembered event) are given in various modalities of experience (including modalities of being, such as actual and potential, possible and certain, real and fictional, and temporal modalities such as past, present, and future). Intentional analysis, thus, lends itself to two-sided *correlational analysis* between subjective modes of consciousness and intentional objects as their correlates (Husserl, 1950, p. 74–75, 1976, §97).

On the objective side of reflective investigation belong not only the singular objects and states of affairs as they are actually attended; it also entails their background as something implicitly, indeterminately, and potentially co-intended. Husserl extended his noematic analyses to (un)cover these background features of experience or *horizons* as he prefers to call them (see e.g., Husserl, 1950, §20, 1976, p. 188–189). What makes it blatantly clear that he does not view phenomenological reflection as restricted to any kind of mental or internal dimension is that Husserl thinks that the world is the ultimate horizon for every single experience. For Husserl, the world is not merely the spatio-temporal background of every perception and other object-directed intentional acts. It is also the practical and social context of everyday activities imbued with familiarity and historical meaning (Husserl, 1939/1972, p. 49, 52–54, 1950, p. 75, 1954, p. 267). This is why Husserl analyzed the subject of experience as a person embedded in and in relation to its temporal, social, and worldly horizons (see Belt, 2019). This is in stark contrast with the narrow-psychological investigation of subjects isolated from the historical, practical, and social world promoted by methodological solipsism.

Rather than limiting phenomenology to intramental contents, intentional analysis widens the scope of reflective investigations. As Husserl stresses, in phenomenological reflection, understood as intentional analysis, “all occurrences of the life turned *toward the world* […] become accessible to description” (Husserl, 1950, p. 73–74, emphasis added). In intentional analysis Husserl puts into effect, through reflective practice, the methodological change brought about by *epoché*. To repeat, this move should not be understood as turning away from the world and leading back to some kind of internal domain, as commentators interpreting Husserl as a methodological solipsist often claim (see McIntyre, 1988, p. 58–59; Varela et al., 1991/2016, p. 16). Neither should it be framed as *constructing* a subjective world from within nor as *postulating* a sort of fictional parallel world by an external observer, as Dennett(1987, p. 154) would have it. Rather than losing the world, Husserl argues, intentional analysis treats the world as the correlate of all possible conscious experience (Husserl, 1939/1972, p. 46, 1950, p. 75, 1954, p. 235–236). From the perspective of transcendental reflection, intentional analysis is executed as a systematic *constitutional analysis* of all the actual and possible ways of being conscious of objective unities in different categories and regions of objects. This delineation opens up constitutional investigations of different levels of objectivity from psychophysical nature to (inter)personal human reality, regions of value and practical objects, cultural formations like “*state, law, morals, and the church*” (Husserl, 1976, p. 354), ontologies of all the different sciences, and ultimately the whole of what can be called the objective world (Husserl, 1950, p. 85–86, 89, 98, 1976, §80, §86, §149, §§152–153). These phenomena are not only intersubjectively accessible, but also non-private in the more elementary sense of being intersubjectively constituted, i.e., an intentional accomplishment based on and mediated by social interaction (rooted in empathy in particular), communalization, and, in many cases, historical formation over generations^32^.

This brief description has hopefully shown that Dennett’s interpretation of Husserl as committed to methodological solipsism runs counter to both how Husserl himself views his method and how reflection as intentional analysis is executed. In other words, Husserl is committed to methodological solipsism neither as a research *strategy* nor as an *implemented* method. Consequently, Dennett misses the positive potential of intentional analysis for alleviating the empirical worries raised in the previous sections, most notably underdescription (our tendency to overlook certain features of conscious experience). Let us end this section by returning to Dennett’s demonstrations of the allegedly undetected defects in our peripheral vision. Interestingly, Husserl elucidates the indeterminacy and vagueness of experience precisely with concrete examples of visual perception in a series of intentional analyses.

Consider looking at a sheet of paper on the desk in front of you. When you are turned toward the paper, you also perceive objects surrounding it: books, pencils, a cup, etc. As long as you stay focused on the paper, however, it is picked out from its perceptual background. While the surrounding objects also appear as something co-given, they are not perceived attentively but only seen in the background with relative degrees of clarity and saliency. Still, you are at any time free to turn your gaze (*Blick*) from the currently regarded object (e.g., the sheet) to any of those background objects in your visual field (e.g., the teacup on the table) and notice it in passing or concentrate upon it. In shifting focus, you become explicitly conscious of another object and its distinctive features, which were only implicitly seen as potential objects of attention. But at the same time the original act of perception directed at the paper loses its actuality and intuitive clarity and the previously attended object recedes into background (Husserl, 1976, §35, §§44–45, §83). Even such an elementary analysis shows that Husserl did not consider the visual field uniformly detailed or fully determined. Rather, what is actually seen is always accompanied or surrounded by what Husserl, in different passages in *Ideen I*, calls *horizons* of “background inattentiveness” (Husserl, 1976, p. 185–186) and “more or less vague indeterminacy” (Husserl, 1976, p. 91), and “‘a halo’ of non-actualities” (Husserl, 1976, p. 73).

Now consider Husserl’s other well-known example, looking at a tree in a garden through a window (Husserl, 1976, §97, cf. §41). While continuously observing the tree, its manners of appearance may change in various ways: the tree itself might sway in the wind or you can alter your own position in relation to it by tilting your head or taking a step closer to the window to get a better view; you can keep your focus fixed on the tree or let your eyes wander to its branches and trunk; finally, the color of the tree is displayed in a wide range of shades in the changing light. Still, throughout these changes, you observe one and the same tree with prevailing and identical features (such as shape but also color). How do we make sense of this phenomenologically? The first step of intentional analysis is to distinguish how the hyletic moments (e.g., kinesthetic sensations, sensations of color), noetic acts (e.g., perceiving, focusing), and noematic aspects (e.g., the tree as it is seen, the perceived color of its trunk) are correlated in visual experience. Then, constitutional analysis sets out to explicate how a continuous and unified consciousness of an unchanging intentional object or “a synthesis of identification” in Husserl’s words is formed in the course of constantly changing perception (Husserl, 1954, p. 160–161, 164).

Husserl’s constitutional analysis focuses, first, on the temporality of visual experience. What is currently seen retains the previous phases of perception and anticipates its future course. Only in this way can I see that the momentary changing perspectives are aspects *of this* tree or shades *of this* color; otherwise I would not see the *same* tree if I closed my eyes for a second or be surprised if the tree turns out be a prop when seen from another angle. Husserl also underlines the embodied aspects of seeing and perceiving in general. One’s lived body is involved in visual perception both as a center of orientation (the tree is perceived in particular direction, near or far, over there in relation to here, etc.) and as a *locus* of action. Visual experience is not only supported by more or less automatic processes (e.g., my eyes saccade and accommodate distances in tracking the swaying branches), but it also entails an implicit or more explicit sense of motility and awareness of possible lines of voluntary action – a consciousness of “I move” and “I can” in Husserl’s terms. These dynamic aspects of perception point to yet another kind of indeterminacy in visual experience and perception in general. Since a tree is always seen from a certain spatial perspective and, thus, presented one-sidedly, it is always open to *further* determinations (there are currently unseen aspects, unnoticed details, etc.) and, correlatively, potential courses of perceptual action: I can *deliberately* change focus or *let* my eyes wander aimlessly, *freely move* around the tree, or force myself to *stay still* and *fix* my eyes more closely on a currently visible detail and so on (Husserl, 1950, §§17–19, 1954, p. 160–161, 164, 1976, §41, §44).

Classical phenomenologists like Husserl and Merleau-Ponty have treated the indeterminacy of vision as an essential feature of perception analyzable in greater detail, not as a contingent cognitive defect. Drawing from these sources, Thompson et al. (1999) demonstrate this by dissecting another of Dennett’s examples, namely the thought experiment where one imagines entering a room covered with a wallpaper made out of identical pictures of Marilyn Monroe. They point out several ways in which Dennett mis- and underdescribes the phenomenology of visual perception (see also Printz, 2018; cf. Dennett, 1991, p. 354–355, 359–360).

First, we do not *seem* to see, as Dennett falsely claims, hundreds of Marilyns equally well and in detail. On the contrary, the pictures currently in front of you are seen clearly while posters get less distinct and eventually indistinguishable further to the sides. This is not something undetected at the personal level, as careful phenomenological descriptions offered in terms of horizons and figure-ground structure show. Second, Dennett’s depiction of the perceptual situation is artificially static and passive, considering the set-up. In stepping into the room, one has already scanned the environment and, as an active perceiver, is always free to explore it in further detail. Thus, one perceives the room covered with Marilyns, not because each poster presents itself as seemingly distinct at the moment but because the pictures not currently focused upon are still co-present as previously seen copies (that have become indistinct in turning one’s head) or potential objects in the future course of perception (that are anticipated but not yet determined). Third, Dennett overlooks the embodied character of visual experience and its spatial configuration: all perceivable things are situated in relation to one’s body and its spatial vantage point. What we are facing and where our gaze is focused on partly determine which Marilyns stand out from the background. Moreover, the visual surroundings can be further divided into the immediate spatial context (the more distinct pictures close to the central figure) and the periphery with indeterminate boundaries (it is not easy to tell which posters fall outside of the visual field). In line with Husserl, Thompson et al. (1999) emphasize that the indeterminate background also involves a tacit awareness of one’s body (and bodily abilities, I might add, epitomized in the “I can”); every perceivable object is situated in what they, following Merleau-Ponty, call “an implicit bodily space.” Along these lines, one could argue that in order to experience the room *covered* with Marilyns, even the surroundings outside of the visual field must be marginally present in perception as something implicitly there. After all, it would surprise us if we turned around to inspect the wall behind our back and the wallpaper looked totally different.

The lessons learned also apply to Dennett’s experiment with playing cards, discussed above. When you are asked to stand still and stare straight ahead at a fixed target, it is certainly easier to isolate certain features of central and peripheral vision (or foveal and parafoveal vision) and notice their limitations. Such an illustration may well reveal everyday misconceptions about visual experience and even point out a common blind spot of casual reflection. But it ought to be taken as a prompt for further phenomenological reflection rather than as an indication of unsurpassable errors of reflection, let alone as proof that such phenomena are indescribable from the first-person perspective. The intentional analyses sketched above can shed light on what is ordinarily overlooked, but they also suggest that Dennett’s examples of peripheral vision pay insufficient attention to temporal, embodied, and (en)active aspects of visual *experience*. They illustrate how Husserlian distinctions and lines of investigation can lead to a more comprehensive and faithful description of previously underdescribed phenomena.

### Eidetic Variation

Dennett’s remaining methodological arguments for phenomenological skepticism all revolve around the question of how phenomenological reflection is related to a single reflecting subject. Arguments stemming from the documented problems of introspective psychology and the perceived deficiencies of introspection in general implicitly assume that Husserlian methodology relies heavily on introspection, i.e., self-observation and the reporting of one’s own current or recently past experiences. Along the same lines, Dennett maintains that autophenomenologists treat *oneself* as the sole subject and only object of investigations (the lone-wolf approach) and base their insights exclusively on what can be learned reflectively from one’s *own* experience (the first-person-plural presumption). This section aims to undermine these assumptions by setting straight the relationship between phenomenological reflection, the reflecting individual, and her particular experience. This is established by taking a closer look at the eidetic nature of Husserl’s reflective phenomenology, especially the methodological procedure called *eidetic variation*. Finally, I will revisit two skepticism-inducing topics of the previous sections, namely cataloging experiences and variation in self-reports.

For Dennett, the perceived inability of introspective psychology to meet the standards of objective science points to a deeper issue inherent in all first-person methods. Since introspection and, by extension, autophenomenology rely on *private* inspection of *one’s own particular* experiences, the argument goes, one can never hope to produce reliable results that can be compared, validated, and replicated intersubjectively using first-person methods. Dennett also seems to agree with William James in thinking that introspection always involves *retro*spection. This poses another problem for reflective methodology. As accessing and self-reporting experiences takes time and proceeds in stages, there is always a logical chance of error due to misremembering, no matter how short the time lapse between experiencing and describing it (Dennett, 1991, p. 317–318; cf. James, 1890/1950, p. 185, 189–192).

Husserl was by no means unfamiliar with such skeptical arguments. A manuscript drafted on Husserl’s behalf by his assistant Edith Stein to address their contemporary psychologists, Theodor Elsenhans and August Messer, even seems to endorse a similar train of thought:

“What is genuinely psychic […] cannot be treated in the same way as external objects. A perception, a feeling of joy, a simple sensation, flows away; and once it has decayed, it has *irretrievably* [*unwiederbringlich*] disappeared. I cannot hold on to them and inspect [*vorzeigen*] them, so as to give some determinacy to the fluid descriptive concepts corresponding to them; I cannot hold them up to each other, so as to isolate common attributes and, with their help, to form classificational concepts. […] I have a flux of unrepeatable [*unwiederholbaren*] and incomparable [*unvergleichbaren*] individualities, which mock any kind of conceptual grasp. *A pure empirical science* [*Erfahrungswissenschaft*] *of the psychic is utterly impossible*.”^33^

Husserl scrutinized the limits of attaining reflective knowledge about the particularities of consciousness also in his published writings. Due to their flowing and fluctuating character, he argues in *Ideen I*, individual experiences can never be completely perceived and fully grasped in reflection; nor can I inspect *my* stream of consciousness in its entirety in the present moment by “swimming after” it retrospectively (Husserl, 1976, p. 93–94, 96, 156–157). How can phenomenological reflection and phenomenology as a discipline, then, claim to overcome these problems?

The basic idea of eidetic phenomenology is simple enough. From an empirical-psychological standpoint, Husserl(1984, p. 6–7, 12–13) maintains, experiences are perceived and treated as particular facts, classes of real events, mental or psychological attributes or the like. Husserl proposes a methodological re-orientation or a change of perspective called *eidetic reduction*, which leads phenomenology to consider experiences according to their essential features and necessary connections (pure essences or *eide* in Husserl’s terminology) (Husserl, 1976, p. 6, 8). Instead of trying to document currently ongoing or previously had individual experiences in an attempt to establish inductive generalizations and empirical classifications, phenomenological reflection sets out to uncover, intuitively apprehend, and analyze the essences of different kinds of experience and their essential relations on different levels of generality and specificity. That is, eidetic investigations focus not only on experience in general as the highest genus, but also on perception, remembering, willing, empathy, etc. as its subordinate kinds or species (Husserl, 1976, p. 30, 157). In short, Husserl presents an alternative to introspective and experimental psychology of his time by developing phenomenology as an eidetic science or a “science of essences” (*Wesenswissenschaft*).

How does eidetic phenomenology examine its subject matter? Husserl(1976, p. 13, 15–16, 69) believes that essences can be intuitively exemplified with both actual and possible instances, no matter if they are currently perceived, remembered or “merely” imagined. Conversely, one is always free to shift focus from a (real or imagined) particular experience or an experience of something singular to corresponding essences in an act called *ideation*. Husserl often illustrates his eidetic method with simple exemplary analyses of perceptual phenomena (such as perceiving a table, hearing a sound, or seeing colors), but ideation is in principle applicable to all kinds of objects of experience from spatial shapes to social processes (Husserl, 1976, §4; see also Husserl, 1950, §34, 1962, §9a,e, §10). Husserl further assures that ideation (or “seeing essences”) is nothing mysterious or metaphysically compromising; rather, it is familiar to anyone who has learned mathematics and actively acquired first-hand geometrical insights (in intuitively determining, for instance, that circle is a type of conic section) (Husserl, 1962, p. 85, 87, 1976, p. 47–48). Pushing the analogy further, he holds that eidetic phenomenology demands working freely in imagination like a geometer who produces figures in phantasy, reshapes them at will, and runs through any number of conceivable configurations to probe geometrical insights (Husserl, 1976, §4, §7, §70). To preserve the freedom and presuppositionlessness of eidetic research, ideation strives to discern essences *purely*, that is, without positing the actuality of their corresponding particulars. The universality of eidetic claims is, therefore, not restricted to actual cases or to what is factually possible (as is the case with empirical generalizations); they extend to all *conceivable* experiences or “pure possibilities” in Husserl’s vocabulary (Husserl, 1939/1972, §82, §86, §§89–90). How is such a transition from particular experiences and empirical generalities to intuitively apprehended pure essences and essential structures supposed to be accomplished reflectively? What is the explicit methodic form of ideation?

As an eidetic method, ideation can be articulated as a procedure called *eidetic variation^34^*. The process involves using imagination and it proceeds in stages. One starts with an actual, remembered, or imagined example considered as an instantiation of a certain type of experience. Taking it as a guiding model, one then modifies its features freely and as far as possible in imagination in order to produce an open-ended series of *variants* of the same type. Running through all the different variations, one is finally supposed to be able to discern and single out their overlapping or coinciding features and to obtain an intuitive grasp of what stays *invariant* throughout the series. This is what Husserl calls *eidos*, pure or universal essence, and necessary or universal form – in short, it is something without which the experience in question is inconceivable. After grasping or “seeing” essences in this way, the resulting eidetic findings can then be conceptualized, further analyzed, and expressed in the form of universal statements or “eidetic laws” in Husserl’s terms.

The previous subsection already provided eidetic descriptions at the highest level of generality. Intentionality was presented as an essential feature of conscious experience in general and further divided into its noetic, hyletic, and noematic moments and horizon structures. These basic distinctions were then applied, by way of example, to concrete cases in intentional analyses focused on the indeterminacy of visual experience. Another essential feature of consciousness touched upon is its temporal structure. Not only are intentional objects of experience constituted as something “fixed and abiding” over time and through changing experiences, but also consciousness itself is temporally constituted (see Husserl, 1950, §20). A melody, for instance, is a temporal object with duration, but our conscious experience of it endures as well. To hear an array of sounds *as* a melody requires that past tones are somehow retained (as just passed) and a succession of chords is implicitly anticipated (as soon to arrive) in what is heard in the current moment; a certain note can appear as a discord only in contrast to such a concordant continuum. Husserl considered it an essential feature of consciousness that each phase of experience has a threefold structure of primal impression, retention, and protention, which unifies conscious experience passing from one now-moment to another and gives a flowing character to it. By virtue of such temporal form, individual episodes have duration (e.g., a feeling of joy initially rises, intensifies, and dissipates in phases, and eventually fades into past), but it also binds conscious experiences together, and this connection of experiences is temporally ordered into successive and simultaneous experiences (Husserl, 1966, p. 66, 72–73, 87, 313–317, 323–324, 1976, §§81–82). This is why Husserl can ultimately claim that “*conscious life as a whole* […] *is synthetically unified*” (Husserl, 1950, p. 80) and that each experience belongs to a “*single* endless ‘*stream of experience’*” (Husserl, 1976, p. 182).

The topic of inner time-consciousness as a basic structure of experience and the fundamental form of synthesis led Husserl to notoriously difficult in-depth explorations whose technical intricacies need not concern us here. The fact that Husserl investigated intentionality and temporality as universal *structures* of conscious experience is already sufficient to demonstrate that eidetic descriptions are by no means restricted to *eide* of different categories and regions of *objects* and their essential features. Correlational and constitutional analyses also strive to explicate eidetically how the subjective and objective sides of experience are necessarily connected and how experiences are *inter*connected in a structured, lawlike manner.

As an eidetic method, phenomenological reflection circumvents the perceived main weaknesses of introspection. First, eidetic phenomenological description is by no means restricted to what is experienced here and now. Neither does it rely on trying to faithfully retrieve already passed and irrevocably faded individual experiences. Since the same essential and structural features are instantiated by countless examples, one can replicate the process of eidetic variation by finding another starting exemplar, producing a new series of variants and (re)evoking eidetic insights^35^. Second, the results are not incommensurable, because one is not reporting individual or private events. Rather, eidetic descriptions are concerned with shared structures of experience and they claim universal validity. They can, therefore, always be compared to and challenged by competing descriptions in terms of clarity, accuracy, scope, amount of detail, ability to differentiate, etc. Third, in arriving at eidetic claims, phenomenological reflection does not simply draw from one’s own experience and (over)generalize. On the contrary, confining eidetic variation to what the reflecting individual has experienced first-hand would seriously constrain our ability to probe what is conceivable. Since our imaginative abilities are also limited, Husserl advocates “pollinating” imagination with experiential, historical, and even fictional sources depicting lived experience before engaging in eidetic investigations (see Husserl, 1952, p. 51–53, 1976, §70). Enriching our imagination with an array of intuitive material to work with extends the scope of variation to unthought-of but still conceivable possibilities and illuminates unnoticed or underdescribed features of conscious experience. One could even argue that this addresses one of Dennett’s recurrent worries, namely that philosophers have a tendency of “mistaking a failure of imagination for an insight into necessity” (Dennett, 1991, p. 401, cf. 48, 440).

Dennett’s conception of autophenomenology fails to recognize the eidetic aspect of phenomenological reflection. This omission is echoed by what is missing from his heterophenomenological alternative. In Dennett’s reading, the goal of autophenomenology is to characterize one’s *own* notional world introspectively “from the inside.” By the same token, heterophenomenology sets out to describe the notional world of *another* subject “from the outside” by interviewing and observing them. In both cases, one starts by extracting a *single* person’s account of their own experience (see Dennett,1987, p. 153, 158; cf. Dennett, 2003, 2007). But how do we generalize from such accounts? Dennett accuses autophenomenologists of carelessly extrapolating from their own experience and simply assuming that the same first-person descriptions are reproducible by others. In heterophenomenology, by contrast, test subjects are deliberately steered away from theorization and “faux generalization” (see Hurlburt and Schwitzgebel, 2007, p. 127, 255) when giving their subjective accounts. It follows that individual reports are only subsequently compared, interpreted, and cataloged. Yet, Dennett says little about how one extrapolates *post facto* from a set of heterophenomenological texts. He is fast to deny that first-person data is “averaged out” by statistical means (Dennett, 2007), but how should one draw conclusions if and when variation between subjects ensues? Dennett (2003, 2007) rightly emphasizes the role of *interpretation* already present in identifying first-person reports, turning them into useful data, and finally using them as a source of evidence. But his remarks on the requirements of interpretation are ambiguous at best. What background knowledge does a researcher need, and is entitled to use, in interpretation? Do heterophenomenologists apply the same vocabulary as test subjects or adopt another terminological or theoretical framework for interpretation? How are the findings ultimately classified?

These are pressing questions, not least because heterophenomenology is supposed to provide a neutral inventory of phenomenological items or a “heterophenomenological catalog” – something Husserl’s phenomenology is supposedly unable to deliver. Dennett(1991, p. 45–46) is the first to admit that his own provisional classification of inner, outer, and affective experiences is based on “dubious tradition” and “superficial similarities” rather than a “deep kinship” between phenomena. Presumably he favors heterophenomenological reports that can be interpreted indirectly and antecedently so that one can refrain from committing to pre-established categories. However, it is unclear how the heterophenomenological approach could avoid such pitfalls since interpretation of test-subjects’ testimonies relies on the intentional stance of the observer. It is hard to see how identifying, describing, and classifying the contents of other people’s reports is possible without resorting to what the interpreter has learned pre-reflectively from what she has lived through and what she already reflectively knows about her own experience (Gallagher, 1997; Carr, 1998; Marbach, 2007). Following James(1890/1950, p. 194–196), one could also argue that describing consciousness is particularly vulnerable to linguistic influences, since we are prone to use “the vocabulary of outward things” and to suppose substantive entities; we also tend to overlook and misconstrue conscious phenomena due to lack of words and “the dependence of psychology on common speech” (cf. Husserl, 1984, p. 15). Consequently, the less informed one is about phenomenologically attuned and reflectively secured distinctions, the more interpretation is guided by preconceived conceptual categories, associative typifications, folk-psychology, and other potential sources of bias. If some kind of taxonomy of conscious experiences is needed for resolving reflective disagreements, as Dennett insists, Husserlian phenomenology offers an invaluable source.

On this point Dennett has partly conceded to criticism. He admits having previously ignored “data” acquired by reflecting on structures of consciousness from the first-person perspective. Moreover, he is happy to conclude that subtle phenomenological distinctions Husserl, among others, provided can be put to good use in conducting heterophenomenological interviews. Phenomenology can enrich the vocabulary and “tease out” aspects of experience at the personal level (Dennett, 2007). This is a step in the right direction. For all intents and purposes, Dennett here acknowledges that Husserlian phenomenology helps to tackle under- and misdescription of conscious experience with terminological and analytical tools.

For the skeptic, interpersonal and intrapersonal variation in introspective self-reports is indicative of the unreliability of first-person methods (see section “Dennett’s Empirical Arguments”). How does phenomenological reflection fare against the argument from variation? One option is to argue that eidetic phenomenology is not vulnerable to whatever sources of error variation in psychological self-observations might indicate by insisting on the differences in establishing and validating eidetic and empirical claims. Simply put, phenomenological reflection neither relies on cumulative results of personal introspection nor bases its claims on inductive reasoning or statistical inference using data collected from untutored test subjects (or surveying the researchers for that matter). Husserl maintains that since eidetic variation operates freely in imagination, without presupposing or positing the actuality of its examples, it should not be mistaken for “empirical variation” restricted to and constrained by factual cases, let alone required to seek experiential confirmation for its factual basis (see Husserl, 1952, p. 47–48, 51, 54, 1974, p. 218–219, 255, 1976, p. 171–172). It would seem to follow that potential distortions in gathering “first-person data” do not carry over to eidetic phenomenology and observed variation (whatever its ultimate cause) has no bearing on phenomenological reflection.

This line of counterargument, however, oversimplifies the relationship between eidetic and empirical knowledge and misses the potential usefulness of (f)actual variation for eidetic phenomenology. Husserl(1939/1972, p. 423, 426, 1962, p. 71, 74, 86) states clearly that every actual occurrence can be turned into a variant and considered as an example, treating it as one pure possibility among others. It follows that eidetic phenomenology can accommodate empirical material by incorporating it into eidetic variation as starting examples or potential variants. In principle, it makes no difference whether the presumed variation is exposed by first-person, second-person, or third-person investigations, as long as the findings are transformed into intuitively imaginable *possible* experiences. This already shows that neither observed variation nor empirical findings in general should be outrightly dismissed or ignored as irrelevant to eidetic claims. More to the point, eidetic phenomenology can accommodate cases where there *are* good reasons to believe that observed variation points to real underlying differences in how we experience things. Zahavi, among others, has suggested that especially the person-level descriptions of real-life deviations and anomalous cases studied in fields such as psychopathology, cognitive and developmental psychology, neurology and anthropology can both challenge our universalistic eidetic claims (as potential empirical counterexamples) and provide illuminating cases for modifying them. Exceptional cases and human variation in general may, then, prompt phenomenologists to revise and refine eidetic descriptions by “pollinating” imagination and extending the scope of eidetic variation as outlined above (see Zahavi, 2017, p. 151–156).

### Intersubjective Validation

In section “Dennett’s Methodological Arguments,” Dennett’s motivation for methodological skepticism was crystallized in the three requirements of scientificity that first-person methods supposedly fail to meet: publicity or intersubjectivity, reliability, and agreement. Above, I have argued that phenomenological reflection is neither a solipsistic nor an introspective technique. This should clear away the main obstacle for thinking that Husserlian phenomenology cannot meet the standards of publicity or intersubjectivity. Simply put, Husserl’s reflective methodology does not investigate conscious experiences as intersubjectively inaccessible phenomena. On the contrary, the interpretation of *epoché* and intentional analysis defended above shows that the scope of phenomenological reflection is extended to the shared world with its publicly available and intersubjectively constituted objects (rather than restricted to any inner, mental, or private domain). Furthermore, eidetic reflection aims to discover essential or structural features of experience (not facts about any single consciousness or private events). Commentators have also stressed the role of language, shared terminology, and communicative efforts in making phenomenological descriptions public and open to mutual criticism from the get-go (phenomenological descriptions are based neither on private language nor any non-linguistic means) (Sokolowski, 2008; cf. Cai, 2011, p. 126–128, 154–155; see also Zaner, 1973). In this way, the proper domain of phenomenological reflection is, in principle, accessible to everyone. However, in Husserl’s view, phenomenological descriptions claim intersubjective validity also in a stronger sense.

For Husserl, the final validity of phenomenological descriptions does not rest on what Dennett calls lone-wolf autophenomenology. The objectivity of phenomenological results is ultimately decided by an intersubjective communal practice, rather than simply *presuming* that others will agree with a subjective account of mine, yours, or anyone else. Husserl(1950, p. 47) states clearly that only the results that can stand the test of mutual clarification and critique can be deemed “objectively valid.” This lengthy passage from *Ideen I* captures both the requirements and potential benefits of phenomenological reflection carried out intersubjectively:

“If one has acquired the right attitude and fortified it through practice, but, above all, if one has gathered the courage to follow the clear instances of essential givenness in a radically unprejudiced manner [*in radikaler Vorurteilslosigkeit*], untroubled by all the currently circulating and learned theories, then firm results quickly ensue, results that are the same for everyone in the same attitude; there arise substantial possibilities of communicating to others what one has seen oneself, testing [*nachprüfen*] their descriptions, bringing out the unnoticed intrusions of empty verbal meanings, and, through subsequent measuring [*Nachmessung*] in intuition, making known and eradicating errors that are possible here as they are in every sphere concerned with validation.”^36^

According to Husserl, then, the possibility of reaching shared results is opened by (1) adopting the phenomenological attitude, (2) sufficient training, and (3) freedom from presuppositions (all supported by *epoché*) while accepting only what is (4) intuitively given in reflection. What is equally important is (5) sharing one’s findings and (6) testing or verifying other people’s results reflectively in order to (7) identify biases and mistakes and to (8) correct errors. While such a general characterization hardly passes as a step-by-step guideline for conducting phenomenological research, it demonstrates that validating results in a scientific community that shares basic methodology, vocabulary, and research practices is quintessential for Husserlian phenomenological reflection. In fact, Husserl was convinced that real advances in reflective phenomenology required generations of researchers all committed to a shared goal, mutual criticism, and taking over others’ work (see Husserl, 1974, p. 36, 1984, p. 16–17). Naturally, there is no way to guarantee that controversies can always be settled and contradictions solved, but, with the shared methodological *praxis* outlined in this section, finding common ground for handling the disputes is much more typical than Dennett gives Husserlian phenomenology credit for.

As should be clear by now, Husserl sees phenomenological reflection neither as an infallible nor an incorrigible process. In particular, Husserl does not appeal to immunity from error on the basis of what Roy called the “double thesis,” namely that consciousness as a whole is both readily accessible and fully transparent to reflection. This is shown by Husserl’s recurring comments about our inability to ever grasp the particularities of flowing and fluctuating conscious experience in its entirety. Husserl voices similar reservations, for instance, in scrutinizing the indubitability of reflective knowledge of the self^37^. While eidetic methodology seeks to elevate phenomenological reflection from the individual and particular to the essential and structural features through ideation, the eidetic procedure by no means *secures* infallible results either. As a repeatable and open-ended process that can incorporate both challenging and illuminating material, eidetic variation, rather, invites a continuous refining of phenomenological descriptions. This is closer to an ideal of science as a fallible but self-correcting endeavor than to the kind of commitment to infallibility and incorrigibility Dennett ascribes to post-Cartesian first-person investigations. In fact, in *Logische Untersuchungen*, Husserl(1984, p. 15–17) discusses difficulties involved in stating and communicating results in such a way that once-acquired phenomenological insights can be reidentified, tested, and confirmed by others well-versed in phenomenological methodology. Overcoming such obstacles is a prerequisite for conceiving phenomenology as a *scientific* philosophy.

According to Husserl’s guidelines, assessing phenomenological results intersubjectively demands shared methodology and terminology, a certain attitude and training, and a cooperative research community open to mutual criticism. How do such requirements square with Dennett’s claim that phenomenology has failed to come up with “a single, settled method” everyone agrees upon? In this section, I have laid out the basic elements of phenomenological reflection to challenge the perception that Husserlian phenomenology is lacking in methodological foundations. While the methodological features are by no means uncontested even within the phenomenological tradition and their details can certainly be challenged, I do not see how *this sort* of methodological debate would merit Dennett’s wholesale methodological skepticism about (auto)phenomenology. It rather seems that Dennett’s call for methodological *unanimity* turns out to be too strong a requirement. By the same standards, his heterophenomenological alternative would hardly pass as a viable method, as the debates surrounding its nature and general acceptance (see Zahavi, 2007), as well as the above-discussed ambiguities concerning interpretation, generalization and classification, show.

When it comes to the related claim that phenomenological methodology has failed to produce agreement about its results, unanimity presents an equally problematic criterion. As Husserl already argued in response to his contemporary critics, if our reflective insights have to be unanimously affirmed to be considered legitimate, the same standard would render all experiential evidence questionable, since both intuition and *Erfahrung* can and have been appealed to tentatively and even arbitrarily^38^. Revisions occur even in such eidetic disciplines as mathematics and logic, but it hardly undermines the *possibility* of attaining firm results and *ideally* even complete evidence. The pursuit of eidetic knowledge does not imply infallibility nor claim freedom from error in phenomenology either^39^. In *Formale und transzendentale Logik* (1929), Husserl states that the possibility of deception pertains to *every* kind of evidence; even ostensibly apodictic evidence can be annulled by further evidence (Husserl, 1974, p. 164). The point is that it takes more than allusions to disagreements, undecided cases, and occasional errors to establish that phenomenological reflection is an *un*reliable method. Dropping the insistence on full agreement pushes the proponents of phenomenological skepticism to specify what counts as a “sufficient degree” of reliability and objectivity for reflective knowledge and how phenomenological reflection purportedly fails to meet those standards. Anecdotal evidence of “the battle of ‘intuitions”’ simply won’t cut it.

## Conclusion

In this article, I provided arguments to dispute Dennett’s methodological claims that Husserlian phenomenology is committed to introspection, methodological solipsism, the first-person-plural presumption, and the lone-wolf approach. In parallel, I suggested how *epoché*, intentional analysis, eidetic variation, and intersubjective validation serve to alleviate the more empirical worries about overinterpretation, underdescription, and disagreement. I concluded by addressing Dennett’s assumption that phenomenological reflection fails to meet at least three criteria of scientificity, namely publicity or intersubjectivity, reliability, and agreement. What is the outcome of these considerations for phenomenological skepticism motivated by the above-mentioned empirical and methodological reasons?

The strong version of phenomenological skepticism is untenable. It pushes Dennett to adopt metaphysical minimalism and even illusionism, rather than securing the coveted ontological neutrality. The *categorical* version of weak skepticism also loses its appeal, since Husserlian phenomenology is not committed to the doctrine of infallibility and its methodology supports corrigibility in practice. There is simply no reason to question the possibility of reflective knowledge *in general* on the grounds that phenomenological reflection can err and its results are open to modifications.

What about the gradual version of weak skepticism? Husserl explicitly acknowledged the elusive nature of conscious experience and identified many of the problems associated with introspection and casual reflection that motivate weaker forms of skepticism. But what really confirms Husserl’s lack of trust in *untutored* reflection is that he went to such great lengths to hone his methodology in order to safeguard phenomenological reflection against such shortcomings. In light of the methodological considerations provided in this article, it seems premature to conclude, *pace* Roy, that Husserl’s phenomenology, in its original form, cannot tolerate a certain degree of fallibility. In recognizing *and* striving to overcome the limitations of reflection in order to attain reflective knowledge, Husserl’s methodology is, rather, well-positioned to alleviate the worries expressed by gradual weak skepticism.

## Author Contributions

The author confirms being the sole contributor of this work and has approved it for publication.

## Conflict of Interest

The author declares that the research was conducted in the absence of any commercial or financial relationships that could be construed as a potential conflict of interest.

## Footnotes

1 Cited in James(1890/1950, p. 188).

2 Roy(2007, p. 4, cf. 9). The term itself is somewhat misleading, since skepticism in question is neither *based on* phenomenological findings nor *driven by* phenomenological attitude (in the sense that it is customary to speak of Pyrrhonian skepticism or Cartesian doubt, for instance). In contrast, it is skepticism *about* first-person methodology and its ability to reliably describe what Roy calls “phenomenological properties” and what Dennett(1991, p. 45) dubs as “phenomenology” (with a lowercase p), namely “the various items in conscious experience that have to be explained.”

3 Carr (1998) offers a more pessimistic view: due to lack of knowledge of phenomenological tradition and insufficient understanding of Husserlian methodology, Dennett ends up adopting elements of Husserl’s reflective methodology without acknowledging it.

4 See Dennett(2007, p. 267): “I am happy not just to concede but to insist that many of the brilliant reflections of Husserl and Husserlians ought to be exploited to the full in heterophenomenological research. I just want to strip them of the anti-naturalistic ideology that has – for the most part – weighed them down […] we can salvage all the good ideas of Phenomenology and incorporate them into heterophenomenology.” Cf. Dennett, 1978, p. 184.

5 For more detailed arguments, see Zahavi(2007, 2017, p. 10–11, 16–17, 25–27, 123–126, 131, 144–150, 163); Cerbone (2003, 2012).

6 Zahavi(2007, 2017, p. 9, 14–15, 27) and Cerbone (2003, 2012) provide both textual evidence and methodological reasons as to why Husserl adamantly distinguished his method from introspection. By contrast, Gutland (2018) argues that Husserl’s methodology can be seen as a refined and systematized kind of introspection, which avoids some of its shortcomings.

7 In the introspection debate, Peels (2016) defines empirical (scientific) arguments as arguments “somehow based on empirical scientific research.” He evaluates five main arguments against the reliability of introspection in the current literature by looking case-by-case at whether the conducted experiments actually support the conclusion. In this article, my goal is simply to reconstruct the empirically motivated general arguments and lines of thinking behind Dennett’s phenomenological skepticism, not to evaluate the actual or potential empirical support for his claims.

8 Dennett(1991, p. 53–54, 68, 2001/2018); cf. Schwitzgebel(2011, p. 125–126). In empirical research, the phenomenon is often divided into distinct but closely related forms of inattentional blindness and change blindness (see again Peels, 2016).

9 The well-known case study by Nisbett and Wilson (1977) has been taken to show that we, in fact, retrospectively posit, rather than introspectively observe, some key factors in our experience, even when we think we are only consciously reflecting. In Dennett’s view, however, our tendency to fabulate extends well beyond the causes of our experiences and the self-attribution of reasons for action examined by Nisbett and Wilson (see Roy, 2007).

10 As Schwitzgebel(2011, p. xi) notes, in addition to cross-cultural variation in reports, differences are found between individuals in the same cultural context *and* within one and the same individual over time. Dennett seems to concur: it is safer to assume that we are all more or less alike but that we err in describing our experiences because of the unreliability of introspection (cf. Dennett, 1991, p. 67).

11 Schwitzgebel(2011, p. 126) also brings up peripheral vision as a case where untutored reflectors are, at first, usually badly mistaken, but can acknowledge their error when warned and trained.

12 Schwitzgebel(2011, Ch. 1) points out that the question “Do we dream in color?” has produced vastly different answers in different times, suggesting that we may not be as firmly convinced as we think even on a subject that should be almost trivial. In Dennett’s case, referring to the unresolved debate about imageless thought in introspective psychology might qualify as a sketch for an argument from uncertainty.

13 Here, I follow Piccinini’s (2010) reading of Dennett.

14 Conversely, Dennett (2003, 2007) argues for his version of *hetero*phenomenology as an extension of science by stressing its conformity to the objective standards of natural sciences and its continuity with existing (reliable) practices in cognitive science, psychology, and scientific study of consciousness. He seemingly consents to his critics in saying that heterophenomenology could have easily been *labeled* as “the second-person method of gathering data” or even “first-person science of consciousness” (Dennett, 2007, p. 252, cf. 263–264). But what are the implications for objective third-person science if it is founded on interpersonal research practices, i.e., studies conducted from the embodied first-person or second-person perspective (or based on intersubjectively constituted life-world, to use Husserlian terms)? Such questions do not prompt Dennett to reform his conception of standard science or to refine the dichotomy of first-person and third-person perspective.

15 Goldman (1997) traces such requirements back to a long line of philosophers of science defending the publicity of science as the core principle of any scientific methodology from Boyle to the positivists, Popper, and Hempel.

16 Dennett(1991, p. 70). The skeptical consensus against first-person accounts was not as unified as Dennett’s testimony of that time might suggest. See Velmans (1991) for the then ongoing debate on the mutual irreducibility and complementarity of the first-person and third-person perspectives in studying consciousness (and related phenomena) amongst empirically oriented psychologists and cognitive scientists. For a recent methodological discussion in the interdisciplinary field of integrative human neuroscience, see Kotchoubey et al. (2016).

17 Dennett(1987, p. 134–135, 141, 153–154). Dennett adopts the term “methodological solipsism” from Hilary Putnam but in *The Intentional Stance* he focuses on Fodor’s (1980) formulation of it. For the perceived affinities between Husserl and Fodor as methodological solipsists, see Dreyfus (1980), McIntyre (1988).

18 By the intentional stance, Dennett means a strategy of treating the object of investigation as a rational agent whose behavior can be predicted in terms of beliefs, desires, and other mental states characterized by intentionality or aboutness (see Dennett, 1987, p. 15, 24, 31–32, 1991, p. 76–77). While using this strategy is usually habitual and effortless, Dennett underlines that the intentional stance adopted freely in heterophenomenology differs from normal interpersonal relations: instead of trusting other subjects’ word on their own experience, one is to maintain “constructive and sympathetic neutrality” (Dennett, 1991, p. 83) by neither challenging nor accepting the veracity of their assertions (see also Dennett, 1991, p. 81, 2001/2018; Zawidzki, 2018).

19 Dennett(1991, p. 71, 459). By no means does Dennett shy away from developing and *eventually* adopting eliminativist and reductionist positions on certain issues. He simply does not want to embrace such views as a *starting point.* See Dennett (2007).

20 See Dennett(1988, 1991, p. 126, 132, 390, 403, 454–455, 459–462). In his debate with David Papineau (see Crane, 2017), Dennett wavers between eliminativist and revisionist formulations in motivating his project of naturalizing consciousness: “It has been my work for fifty years to provide alternative, naturalistic alternatives to all these chimeras; it is not that consciousness doesn’t exist but that it isn’t what you probably think it is. (Qualia are a theorist’s illusion but the subjectivity of experience is real.)”

21 Dennett now accepts a distinction he earlier disavowed, namely the dichotomy between the way things (actually or objectively) seem to you as opposed to how they *seem* to seem to you. See Dennett (2018) commenting on Rosenthal (2018), cf. Dennett(1991, p. 132).

22 Schwitzgebel (2007) has pointed out that Dennett, for instance, claims there is no *actual* phenomenology while *not* wishing to deny that conscious experience is something real. He also holds that we are never infallible about our own conscious experience while granting us limited incorrigibility – the last word – on our reports how it *seems* to us or “what it is like to be us.”

23 Gallagher (2003) distinguishes three such approaches: neurophenomenology, front-loaded phenomenology, and indirect phenomenology. For *neurophenomenology*, cognitive neuroscience and phenomenological analyses are related through mutual constraints and advanced reciprocally (Varela, 1996). This involves training both scientists and test subjects in phenomenological practice to elicit refined first-person descriptions and descriptive categories, which are then used to analyze the data correlating with third-person behavioral and brain activity measurements (Gallagher, 2003). Early neurophenomenological case studies already used open questions in formulating descriptive categories (Lutz, 2002), but second-person interview techniques have since been refined in the neurophenomenological context and further developed into a novel approach called *micro-phenomenology* (Petitmengin, 2006, 2010; Petitmengin et al., 2019). *Front-loaded phenomenology*, in turn, utilizes the analytical tools and insights of phenomenological research in designing experimental setups and interpreting their results, without having to rely on training test subjects or requiring introspective reports. Finally, *indirect phenomenology* applies phenomenological insights to (re)interpret experimental results retrospectively. As Gallagher notes, indirect use of phenomenology eventually reraises the questions of how to use the phenomenological method *directly* and how to incorporate its result into the experimental context.

24 Husserl(1976, §78). Several commentators have pointed out that this transformative modification is instrumental in attaining reflective knowledge (see Thomasson, 2003; Zahavi, 2015).

25 Husserl(1984, p. 15) himself acknowledges the same problem.

26 One could argue that in the last three decades Dennett has indeed reflected on the place and function of human consciousness and intentionality in the natural world by presenting a (naturalistic) theory of their emergence and gradual evolution by natural selection. In his efforts to integrate them into the scientific worldview, Dennett has openly committed himself to Darwinian adaptationism, granting neither phenomenal consciousness nor intrinsic intentionality a role in the evolution of human cognition (see Zawidzki, 2007, p. 81–85, 122, 126–129; Thompson, 2009, p. 4, 6, 70, 74). Still, this leaves the status of the natural-scientific worldview, its reality and objectivity, unclarified and offers little justification for adopting it. Thompson has suggested that a more comprehensive application of *epoché* and Husserl’s notion of constitution could push Dennett to make his “scientific realism” consistent with his analysis of consciousness and theory of stances, for which the reality of objects seems to depend on the adopted stance (Thompson, 2000, 2009, p. 98, 103–105, 152–154).

27 For these two lines of interpretation of *epoché* and their problems, see Zahavi, 2019.

28 It is outside the scope of this article to determine whether Dennett’s passing remarks on Brentano are any more faithful to the original than his reading of Husserl. I will not take a stand on the accuracy of Husserl’s reading of Brentano either.

29 In contrast to Brentano (and Husserl), Dennett’s intentional systems theory decouples intentionality from phenomenal consciousness. In attributing intentional states such as beliefs to others, the intentional stance does not distinguish conscious from unconscious states (Dennett, 2018). Any subject of such intentional attributions whose behavior is reliably predictable from the intentional stance is an *intentional system*, whether it is a human being or other animal, a zombie or a Martian, a corporation or a chess-playing computer (Dennett, 1987, p. 15, 22–23, 28, 58, 1991, p. 76–78).

30 For a recent discussion, see Drummond (2012), Zahavi(2017, p. 10, 52–53, 79–94, 120).

31 In the following exposition, I draw from Moran (2000), Cobb-Stevens (2003), Fisette (2010), and Heffernan (2015).

32 These lines of investigation are already predelineated in such “egological” introductory works as *Ideen I* (Husserl, 1976, §§151–152) and *Cartesianische Meditationen* (in the Fifth Meditation, Husserl, 1950, §43, §49, §58).

33 Husserl(1987, p. 234), Husserl and Stein(2018, p. 455), translation modified; cf. Husserl(1954, p. 181). Reflection being “ever retrospective” was one of the root causes of skeptical concerns raised by yet another contemporary psychologist, Henry J. Watt, whose arguments Husserl(1976, §79) scrutinizes in *Ideen I*. For the parallels between Watt’s and Dennett’s skepticism, see Cerbone(2003 , p. 124–128, 2012, p. 12–16). For Husserl’s response, see also Cai(2011, p. 51–54, 90–92), Zahavi (2015).

34 This reconstruction of eidetic variation is based on Husserl(1939/1972, §87a,e, 1962, §9a,c,e, 1974, §98, 1987, p. 245).

35 Husserl(1974, p. 255, 1976, §75, cf. §7, §34, 1987, p. 234–235, 245). Cerbone (2003, 2012) offers a more detailed answer to Dennett’s argument concerning error from misremembering.

36 Husserl(1976, p. 201, 2014, p. 173), translation modified.

37 Husserl differentiates between (ongoing) experiences, abilities, and (habitual) dispositions as individual contents of the concrete ego that are all accessible to reflection but with different levels of clarity and certainty. Even though the ego’s “living self-presence” (*lebendige Selbstgegenwart*) and its temporal structure might be indubitable, its own past (*Selbstvergangenheit*) is given indeterminately or even obscurely, and its abilities and dispositions are not indubitable regarding their details (Husserl, 1950, p. 61–62, 67). Husserl(1950, p. 62) even alludes to the same kind of skeptical questions concerning self-deception as Dennett: “How far can the transcendental ego [*transzendentale Ich*] be deceived about itself and how far do the absolutely indubitable components extend despite the possible deception?” Knowledge about *what* is indubitable in self-experience and *how far* the clarity of “I am” reaches must be determined critically; knowledge about such questions can only be attained as a “critical achievement” (see Husserl, 2002, p. 401–402; Cai, 2011, p. 110–111).

38 See Husserl(1976, §79) answering psychologist Theodor Ziehen’s skeptical remarks (cf. Cerbone, 2003, p. 128, 132).

39 See again the manuscript addressing Elsenhans and Messer (Husserl, 1987, p. 246–247).
